# Acute hyperglycemia, a rabble-rouser or innocent bystander? A prospective analysis of clinical implications of acute hyperglycemia in STE-ACS patients

**DOI:** 10.1186/s12872-023-03440-3

**Published:** 2023-08-18

**Authors:** Rajesh Kumar, Ali Ammar, Ashok Kumar, Ahsan Ali, Mir Fahad Hussain Talpur, Kubbra Rahooja, Kalsoom Chachar, Anesh Wadhwa, Jawaid Akbar Sial, Tahir Saghir, Sohail Khan, Abdul Hakeem, Nadeem Qamar, Musa Karim

**Affiliations:** 1 National Institute of Cardiovascular Diseases (NICVD), Karachi, Pakistan; 2 Dow University of Health Sciences (DUHS), Karachi, Pakistan

**Keywords:** Acute hyperglycemia, Acute coronary syndrome, Adverse in-hospital outcome, Primary percutaneous coronary intervention, Multi-vessel disease, LVEDP, STE-ACS

## Abstract

**Background:**

Acute hyperglycemia is considered an independent prognosticator of both in-hospital and long-term outcomes in patients with acute coronary syndrome (ACS). This study aimed To analyze the incidence of acute hyperglycemia and its impact on the adverse in-hospital outcome in patients with STE-ACS undergoing primary percutaneous coronary intervention (PCI).

**Methods:**

In this study, we enrolled patients presenting with STE-ACS and undergoing primary PCI at a tertiary care cardiac center. Acute hyperglycemia was defined as random plasma glucose (RBS) > 200 mg/dl at the time of presentation to the emergency room.

**Results:**

Of the 4470 patients, 78.8% were males, and the mean age was 55.52 ± 11 years. In total, 39.4% (1759) were found to have acute hyperglycemia, and of these, 59% (1037) were already diagnosed with diabetes. Patients with acute hyperglycemia were observed to have a higher incidence of heart failure (8.2% vs. 5.5%; p < 0.001), contrast-induced nephropathy (10.9% vs. 7.4%; p < 0.001), and in-hospital mortality (5.7% vs. 2.5%; p < 0.001). On multivariable analysis, acute hyperglycemia was found to be an independent predictor of mortality with an adjusted odds ratio of 1.81 [1.28–2.55]. Multi-vessel disease (1.73 [1.17–2.56]), pre-procedure left ventricular end-diastolic pressure (LVEDP) (1.02 [1.0-1.03]), and Killip class III/IV (4.55 [3.09–6.71]) were found to be the additional independent predictors of in-hospital mortality.

**Conclusions:**

Acute hyperglycemia, regardless of diabetic status, is an independent predictor of in-hospital mortality among patients with STE-ACS undergoing primary PCI. Acute hyperglycemia, along with other significant predictors such as multi-vessel involvement, LVEDP, and Killip class III/IV, can be considered for the risk stratification of these patients.

## Background

Contrary to the high-income communities, an unprecedented increase in the burden of cardiovascular disease (CVD) in low- and middle-income countries (LMICs) is alarming, and it is a massive global problem considering the roughly 80% share by LMICs to the global population [1]. The significant improvements in the management strategy and introduction of primary percutaneous coronary intervention (PCI) in the last two decades resulted in higher survival after ST-segment elevation myocardial infarction (STEMI) [2]. Till it remained one of the leading causes of premature deaths and disability-adjusted life years (DALYs) globally, particularly in low- and middle-income countries [1]. It has direct and indirect financial implications as STEMI tends to affect more working-age people in LMICs compared to high-income communities [3]. Hence, continuous effectors towards improving the management and identification of factors leading to adverse outcomes are crucial.

Among various factors, diabetes mellitus (DM) is a well-established strong independent predictor of major adverse cardiovascular events (MACE) after primary PCI [4, 5]. Acute hyperglycemia on admission, regardless of DM, is also observed to be strongly related to an increased risk of short- and long-term MACE after STEMI [6–8]. It has been reported that around 20 to 50% of the patients exhibit acute hyperglycemia, regardless of DM, possibly in response to physiological stress [9]. However, the exact pathophysiology of acute plasma glucose impairment is poorly explained. Among various postulates for this epiphenomenon, the accumulation of excessive free fatty acids and catecholamine-induced glycogenolysis in response to stress are commonly reported mechanisms [10, 11]. Additionally, this adrenergic and inflammatory response triggered due to ischemia stress, is also found to be associated with diminished collateral circulation, extended myocardial damage, and thrombosis in STEMI patients, consequently resulting in a higher risk of stent thrombosis, no-reflow, life-threatening arrhythmias, and mortality [11–13].

Impaired admission plasma glucose can be either a transient physiologic response to the acute event or due to poor chronic glycemic control; at times, it can be difficult to differentiate between the two [14]. Pre-procedure assessment for acute hyperglycemia is even more relevant in our population as there is evidence of an incidental diagnosis of DM in a significant number of patients, up to 29.6%, admitted with acute myocardial infarction who were not known to have a diagnosis of DM [15]. Therefore, being the largest cardiac care center in the country that provides 24/7 free-of-charge services to cardiac patients, our aim was to evaluate the prognostic role of acute hyperglycemia in the assessment of in-hospital mortality in a much larger and more representative cohort of STEMI patients undergoing primary PCI at a tertiary care cardiac center in Pakistan.

## Methods

### Study population

This is a single-center prospective observational study conducted at the largest cardiac care center in Pakistan, namely the “National Institute of Cardiovascular Diseases (NICVD), Karachi, Pakistan” between January 2022 and June 2022. The ethical review board of the hospital approved the study proposal (ERC-01/2022), and verbal consent was obtained from all the patients or attendants as per the Declaration of Helsinki. For this study, we analyzed prospectively collected data from the cohort of patients diagnosed with STEMI undergoing primary PCI. This contemporary cohort of STEMI patients consisted of adult patients (≥ 18 years) of either gender who shifted to the cardiac catheterization laboratory for primary PCI as per the hospital protocol. Patients who refused to participate in the study were excluded.

### Assessments and definitions

STEMI was diagnosed based on history and a 12-lead electrocardiogram (ECG). Patients with a history of “typical chest pain for at least 20 minutes” and 12-lead ECG finding of “ST elevation in at least two contiguous leads > 2mm in men or > 1mm in women in leads V2 to V3 and/or > 1mm in other contiguous chest leads or limb leads”. Random blood sugar (RBS) was obtained as a pre-procedure assessment for all the patients, and acute hyperglycemia was defined as “RBS ≥ 200 mg/dL”. Patients with a history of clinical diagnosis of diabetes, either on dietary control or anti-hyperglycemic medications (oral or insulin) for the duration of at least six months, were taken as people with diabetes.

### Management and data collection

As per the hospital policy, all the primary PCI procedures were performed free of cost in accordance with standard clinical practice guidelines. All the patients were premeditated with DAPT (dual antiplatelet therapy), which included aspirin and clopidogrel, unfractionated heparin, along with the infusion of glycoprotein inhibitors (IIb/IIIa). Additionally, based on the baseline blood sugar levels, intra-venus (IV) insulin bolus was administered in accordance with the stand insulin sliding scale to hyperglycemic patients. The demographic, clinical, and angiographic characteristics were obtained using a pre-defined proforma. All the patients were observed during their hospital course, and post-procedure complications and outcomes, including mortality, were observed.

### Statistical analysis

Patients were stratified into two groups, normoglycemic and acute hyperglycemic, as defined above. Two study groups were compared in terms of the distribution of demographic, clinical, and angiographic characteristics and post-procedure complications and outcomes. Summary statistics such as mean ± standard deviation (SD)/median [interquartile range (IQR)] or frequency (%), appropriately. Appropriate statistical tests such as independent sample t-test (for normally distributed variables)/Mann-Whitney U test (for non-normal variables), or Chi-square test/Fisher’s exact test was used for the comparison. Considering the significant confounding effect of diabetes, the normoglycemic and acute hyperglycemic groups were also compared within diabetic and non-diabetic contexts. The univariate binary logistic regression analysis was performed for in-hospital mortality for all clinically significant potential predictors of mortality, including female gender, age (years), total ischemic time (hours), Killip class III/IV at presentation, hypertension, diabetes, smoking, history of cerebrovascular accident (CVA)/stroke, obesity (body mass index ≥ 30 kg/m^2^), pre-procedure LVEDP (mmHg), pre-procedure ejection fraction (%), multi-vessel disease, and acute hyperglycemia. Variables with a p-value of < 0.20 in the univariate analysis were taken to the multivariable logistic regression analysis for in-hospital mortality. The odds ratio (OR) [95% confidence interval (CI)] was reported for both univariate and multivariable binary logistic regression analysis. All the statistical analyses were performed using IBM SPSS version 21, and a p-value < 0.05 was considered statistically significant.

## Results

Of the 4470 patients, 78.8% were males, and the mean age was 55.52 ± 11 years. In total, 39.4% (1759) were found to have acute hyperglycemia; of these, 59% (1037) were already diagnosed with DM, making a total of 65.4% of diabetic patients with impaired plasma glucose levels.

Acute hyperglycemia was found to be associated with female gender (25.1% vs. 18.9%; p < 0.001), prolonged ischaemic time (380 [IQR: 260–520] vs. 355 [IQR: 240–495]; p < 0.001), Killip class III/IV at presentation (9.5% vs. 6.1%), hypertension (58.8% vs. 49%; p < 0.001), obesity (18.4% vs. 15.9%; p = 0.030). On angiogram, patients with acute hyperglycemia had higher pre-procedure LVEDP (22.29 ± 10.27 vs. 21.33 ± 9.66 mmHg; p < 0.001), multi-vessel diseases (67% vs. 62%), and culprit right coronary artery (RCA) (32.1% vs. 29.9%). Patients with acute hyperglycemia were observed to have a higher incidence of heart failure (8.2% vs. 5.5%; p < 0.001), contrast-induced nephropathy (CIN) (10.9% vs. 7.4%; p < 0.001), and in-hospital mortality (5.7% vs. 2.5%; p < 0.001) (Table 1).

**Table 1:** Comparison of normoglycemic and acute hyperglycemic patients in terms of the distribution of demographic, clinical, and angiographic characteristics and post-procedure complications and outcomes

	Total	Acute hyperglycemia		P-value
		Normoglycemic	Acute hyperglycemic	
**Total (N)**	**4470**	**60.6% (2711)**	**39.4% (1759)**	
**Gender**				
Male	78.7% (3517)	81.1% (2199)	74.9% (1318)	< 0.001
Female	21.3% (953)	18.9% (512)	25.1% (441)	
**Age (year)**	55.52 ± 11	55.27 ± 11.17	55.89 ± 10.73	0.068
18 to 40 years	10.1% (451)	10.9% (296)	8.8% (155)	0.058
41 to 65 years	74.1% (3313)	73.7% (1999)	74.7% (1314)	
> 65 years	15.8% (706)	15.3% (416)	16.5% (290)	
**Total ischemic time (minutes)**	360 [240–507]	355 [240–495]	380 [260–520]	< 0.001
**Systolic blood pressure (mmHg)**	134.49 ± 24.91	134.21 ± 24.51	134.94 ± 25.51	0.338
**Heart rate (bpm)**	85.94 ± 18.86	84.97 ± 18.1	87.43 ± 19.89	< 0.001
**Random blood sugar**	180 [142–230]	152 [130–171]	245 [220–294]	< 0.001
**Killip Class**				
I	83.8% (3747)	85% (2304)	82% (1443)	< 0.001
II	8.7% (390)	8.9% (241)	8.5% (149)	
III	4.8% (213)	3.8% (104)	6.2% (109)	
IV	2.7% (120)	2.3% (62)	3.3% (58)	
**Co-morbid conditions**				
Hypertension	52.9% (2363)	49% (1328)	58.8% (1035)	< 0.001
Diabetes	35.5% (1586)	20.3% (549)	59% (1037)	< 0.001
Smoking	25.5% (1141)	29.8% (807)	19% (334)	< 0.001
Ischemic heart diseases	9.1% (405)	8.1% (220)	10.5% (185)	0.006
History of CVA/stroke	1.3% (59)	1.1% (30)	1.6% (29)	0.121
Obesity	16.9% (756)	15.9% (432)	18.4% (324)	0.030
**Pre-procedure LVEDP (mmHg)**	21.71 ± 9.92	21.33 ± 9.66	22.29 ± 10.27	0.002
**Pre-procedure EF (%)**	40.19 ± 9.18	40.21 ± 9	40.15 ± 9.45	0.817
**Number of involved vessels**				
Single vessel disease	36.1% (1612)	38% (1031)	33% (581)	0.003
Two vessel disease	34.8% (1555)	33.8% (915)	36.4% (640)	
Three vessel disease	29.1% (1303)	28.2% (765)	30.6% (538)	
**Culprit vessel**				
Left main	0.9% (38)	0.7% (20)	1% (18)	0.031
Proximal LAD	34.9% (1561)	35.2% (954)	34.5% (607)	
Non-proximal LAD	18.8% (839)	19.3% (523)	18% (316)	
Right coronary artery	30.8% (1375)	29.9% (811)	32.1% (564)	
Left circumflex	12.6% (563)	12.8% (346)	12.3% (217)	
Diagonal	1.7% (74)	1.9% (51)	1.3% (23)	
Ramus	0.4% (20)	0.2% (6)	0.8% (14)	
**Pre-procedure TIMI flow**				
0	50.5% (2257)	51.3% (1392)	49.2% (865)	0.439
I	8.9% (396)	8.9% (241)	8.8% (155)	
II	20.6% (921)	19.9% (540)	21.7% (381)	
III	20% (896)	19.8% (538)	20.4% (358)	
**Complications and outcomes**				
Slow flow/no-flow	19.9% (888)	20.6% (559)	18.7% (329)	0.117
Pump failure	6.6% (293)	5.5% (149)	8.2% (144)	< 0.001
Contrast-induced nephropathy	8.8% (392)	7.4% (201)	10.9% (191)	< 0.001
Major bleeding	0.6% (25)	0.3% (9)	0.9% (16)	0.011
CVA/stroke	0.3% (14)	0.3% (8)	0.3% (6)	0.788
Access site complications	0.6% (28)	0.6% (17)	0.6% (11)	0.994
In-hospital mortality	3.8% (170)	2.5% (69)	5.7% (101)	< 0.001

*LVEDP: “left ventricular end-diastolic pressure”, EF: “ejection fraction”, LAD: “left anterior descending artery”, TIMI: “thrombolysis in myocardial infarction”, CVA: “Cerebrovascular accident”*

In non-diabetic patients, acute-hyperglycemia was found to be associated with female gender (20.1% vs. 16.9%; p = 0.055), longer ischemic time (380 [240–504] vs. 355 [240–490] minutes; p = 0.024), raised heart rate at presentation (87.02 ± 18.62 vs. 84.53 ± 17.54 bpm; p = 0.001), Killip class III/IV at presentation (8.3% vs. 5.9%), and elevated LVEDP (22.15 ± 10.58 vs. 21.13 ± 9.61; p = 0.023). A higher incidence of heart failure (7.2% vs. 4.1%; p = 0.001) and mortality (4.0% vs. 2.5%; p = 0.028) among non-diabetic patients was also associated with acute hyperglycemia.

The prolonged ischemia was also found to be associated with acute hyperglycemia in diabetic patients too (380 [270–540] vs. 360 [240–520]; p = 0.023). The acute-hyperglycemia was associated with an increased risk of mortality in diabetic patients, too (6.9% vs. 2.9%; p = 0.001) (Table 2).

**Table 2:** Comparison of normoglycemic and acute hyperglycemic patients, within the context of diabetes and non-diabetes, in terms of the distribution of demographic, clinical, and angiographic characteristics

	Non-diabetics		P-value	Diabetics		P-value
	Normo-glycemic	Acute hyperglycemic		Normo-glycemic	Acute hyperglycemic	
**Total (N)**	**75% (2162)**	**25% (722)**		**34.6% (549)**	**65.4% (1037)**	
**Gender**						
Male	83.1% (1796)	79.9% (577)	0.055	73.4% (403)	71.5% (741)	0.410
Female	16.9% (366)	20.1% (145)		26.6% (146)	28.5% (296)	
**Age (year)**	54.93 ± 11.21	55.3 ± 11.52	0.436	56.64 ± 10.91	56.3 ± 10.13	0.531
18 to 40 years	11.6% (251)	11.1% (80)	0.577	8.2% (45)	7.2% (75)	0.775
41 to 65 years	73.4% (1586)	72.3% (522)		75.2% (413)	76.4% (792)	
> 65 years	15% (325)	16.6% (120)		16.6% (91)	16.4% (170)	
**Total ischemic time (minutes)**	355 [240–490]	380 [240–504]	0.024	360 [240–520]	380 [270–540]	0.023
**Systolic blood pressure (mmHg)**	134.45 ± 24.53	135.61 ± 24.62	0.270	133.24 ± 24.44	134.46 ± 26.11	0.364
**Heart rate (bpm)**	84.53 ± 17.54	87.02 ± 18.62	0.001	86.71 ± 20.09	87.72 ± 20.73	0.350
**Random blood sugar**	150 [128–168]	226 [210–243]	< 0.001	163 [134–181]	264 [234–330]	< 0.001
**Killip Class**						
I	85.1% (1840)	85.3% (616)	0.020	84.5% (464)	79.7% (827)	0.106
II	9% (195)	6.4% (46)		8.4% (46)	9.9% (103)	
III	3.6% (77)	5.4% (39)		4.9% (27)	6.8% (70)	
IV	2.3% (50)	2.9% (21)		2.2% (12)	3.6% (37)	
**Co-morbid conditions**						
Hypertension	42.4% (916)	41% (296)	0.518	75% (412)	71.3% (739)	0.108
Smoking	34.5% (745)	24.5% (177)	< 0.001	11.3% (62)	15.1% (157)	0.035
Ischemic heart diseases	7.8% (168)	9.8% (71)	0.082	9.5% (52)	11% (114)	0.346
History of CVA/stroke	1.1% (23)	1% (7)	0.829	1.3% (7)	2.1% (22)	0.231
Obesity	16% (345)	18.8% (136)	0.072	15.8% (87)	18.1% (188)	0.253
**Pre-procedure LVEDP (mmHg)**	21.13 ± 9.61	22.15 ± 10.58	0.023	22.13 ± 9.86	22.38 ± 10.05	0.628
**Pre-procedure EF (%)**	40.53 ± 8.95	40.15 ± 9.26	0.330	38.95 ± 9.09	40.14 ± 9.59	0.017
**Number of involved vessels**						
Single vessel disease	39.7% (859)	37.5% (271)	0.578	31.3% (172)	29.9% (310)	0.663
Two vessel disease	33.3% (721)	34.6% (250)		35.3% (194)	37.6% (390)	
Three vessel disease	26.9% (582)	27.8% (201)		33.3% (183)	32.5% (337)	
**Culprit vessel**						
Left main	0.7% (15)	1.4% (10)	0.028	0.9% (5)	0.8% (8)	0.430
Proximal LAD	35.1% (759)	38.2% (276)		35.5% (195)	31.9% (331)	
Non-proximal LAD	19.7% (425)	19.4% (140)		17.9% (98)	17% (176)	
Right coronary artery	29.7% (642)	27% (195)		30.8% (169)	35.6% (369)	
Left circumflex	12.6% (272)	11.5% (83)		13.5% (74)	12.9% (134)	
Diagonal	2% (44)	1.5% (11)		1.3% (7)	1.2% (12)	
Ramus	0.2% (5)	1% (7)		0.2% (1)	0.7% (7)	
**Pre-procedure TIMI flow**						
0	51% (1102)	45.3% (327)	0.055	52.8% (290)	51.9% (538)	0.977
I	9% (195)	9.7% (70)		8.4% (46)	8.2% (85)	
II	19.9% (430)	23.4% (169)		20% (110)	20.4% (212)	
III	20.1% (435)	21.6% (156)		18.8% (103)	19.5% (202)	

*LVEDP: “left ventricular end-diastolic pressure”, EF: “ejection fraction”, LAD: “left anterior descending artery”, TIMI: “thrombolysis in myocardial infarction”, CVA: “Cerebrovascular accident”*

Compared to the in-hospital mortality rate of 2.5% for the non-diabetic normoglycemic patients, a significantly higher mortality rate of 4.0% (p = 0.028) was observed for non-diabetic hyperglycemic and 6.9% (p < 0.001) for diabetic hyperglycemic patients, while the in-hospital mortality rate of 2.9% (p = 0.539) for the diabetic normoglycemic patients was not significant from non-diabetic normoglycemic patients (Table 3).

**Table 3:** Comparison of normoglycemic and acute hyperglycemic patients, within the context of diabetes and non-diabetes, in terms of post-procedure complications and outcomes

	*Non-diabetic + normoglycemic	Non-diabetic + hyperglycemic		Diabetic + normoglycemic		Diabetic + hyperglycemic	
		% (n)	P-value	% (n)	P-value	% (n)	P-value
**Total (N)**	**2162**	**722**	**-**	**549**	**-**	**1037**	-
Slow flow/no-flow	20.1% (435)	16.2% (117)	0.021	22.6% (124)	0.202	20.4% (212)	0.813
Pump failure	4.1% (89)	7.2% (52)	0.001	10.9% (60)	< 0.001	8.9% (92)	< 0.001
Contrast-induced nephropathy	6.5% (141)	8% (58)	0.165	10.9% (60)	< 0.001	12.8% (133)	< 0.001
Major bleeding	0.4% (8)	0.8% (6)	0.129	0.2% (1)	0.697	1% (10)	0.035
CVA/stroke	0.3% (6)	0.1% (1)	0.688	0.4% (2)	0.667	0.5% (5)	0.350
Access site complications	0.7% (15)	0.7% (5)	0.997	0.4% (2)	0.550	0.6% (6)	0.706
In-hospital mortality	2.5% (53)	4% (29)	0.028	2.9% (16)	0.539	6.9% (72)	< 0.001

*control group, CVA: “Cerebrovascular accident”

On multivariable analysis, acute hyperglycemia was found to be an independent predictor of mortality with an adjusted OR of 1.81 [95% CI: 1.28–2.55]. Multi-vessel disease (1.73 [95% CI: 1.17–2.56]), pre-procedure LVEDP (1.02 [95% CI: 1.0-1.03]), and Killip class III/IV (4.55 [95% CI: 3.09–6.71]) were found to be additional independent predictors of in-hospital mortality (Table 4).

**Table 4:** The univariate and multivariable logistic regression analysis for in-hospital mortality

	Univariate		Multivariable	
	OR [95% CI]	P-value	OR [95% CI]	P-value
Female	1.26 [0.89–1.8]	0.198	-	-
Age (year)	1.31 [0.97–1.77]	0.080	1.01 [1.00 -1.03]	0.098
Total ischemic time (hours)	1.00 [1.00 -1.01]	0.270	-	-
Killip class III/IV	6.92 [4.91–9.76]	< 0.001	4.55 [3.09–6.71]	< 0.001
Hypertension	1.05 [0.77–1.43]	0.738	-	-
Diabetes	2.01 [1.48–2.73]	< 0.001	1.27 [0.90–1.80]	0.174
Smoking	0.54 [0.36–0.82]	0.004	0.69 [0.45–1.07]	0.094
History of CVA/stroke	2.38 [0.94–6.03]	0.067	1.95 [0.73–5.19]	0.182
Obesity	1.01 [0.67–1.52]	0.959	-	-
Pre-procedure LVEDP (mmHg)	1.03 [1.02–1.05]	< 0.001	1.02 [1.00 -1.03]	0.021
Pre-procedure ejection fraction (%)	0.96 [0.94–0.98]	< 0.001	0.99 [0.97 -1.00]	0.137
Multi-vessel disease	2.23 [1.53–3.26]	< 0.001	1.73 [1.17–2.56]	0.006
Acute hyperglycemia	2.33 [1.71–3.19]	< 0.001	1.81 [1.28–2.55]	< 0.001

*LVEDP: “left ventricular end-diastolic pressure”, CVA: “Cerebrovascular accident”, OR: “odds ratio”, CI: “confidence interval”*

On multivariable analysis, diabetic hyperglycemic patients were found to have significantly higher mortality risk as compared to non-diabetic normoglycemic patients with an adjusted OR of 2.26 [95% CI: 1.54–3.3] as presented in Table 5.

**Table 5:** The univariate and multivariable logistic regression analysis for in-hospital mortality in the context diabetes and acute hyperglycemia

	Univariate		Multivariable	
	OR [95% CI]	P-value	OR [95% CI]	P-value
Female	1.26 [0.89–1.8]	0.198	-	-
Age (year)	1.31 [0.97–1.77]	0.080	1.01 [1.00-1.03]	0.096
Total ischemic time (hours)	1.00 [1.00-1.01]	0.270	-	-
Killip class III/IV	6.92 [4.91–9.76]	< 0.001	4.53 [3.07–6.67]	< 0.001
Hypertension	1.05 [0.77–1.43]	0.738	-	-
Smoking	0.54 [0.36–0.82]	0.004	0.68 [0.44–1.05]	0.083
History of CVA/stroke	2.38 [0.94–6.03]	0.067	1.93 [0.73–5.14]	0.186
Obesity	1.01 [0.67–1.52]	0.959	-	-
Pre-procedure LVEDP (mmHg)	1.03 [1.02–1.05]	< 0.001	1.02 [1.00-1.04]	0.021
Pre-procedure ejection fraction (%)	0.96 [0.94–0.98]	< 0.001	0.99 [0.97-1.00]	0.122
Multi-vessel disease	2.23 [1.53–3.26]	< 0.001	1.74 [1.17–2.57]	0.006
**Acute hyperglycemia**				
*Non-diabetic + normoglycemic	1	NA	1	NA
Non-diabetic + hyperglycemic	1.67 [1.05–2.64]	0.030	1.47 [0.92–2.35]	0.110
Diabetic + normoglycemic	1.19 [0.68–2.11]	0.539	0.94 [0.53–1.69]	0.842
Diabetic + hyperglycemic	2.97 [2.07–4.27]	< 0.001	2.26 [1.54–3.30]	< 0.001

*LVEDP: “left ventricular end-diastolic pressure”, CVA: “Cerebrovascular accident”, OR: “odds ratio”, CI: “confidence interval”*

*control group

## Discussion

On admission, acute hyperglycemia is considered an independent prognosticator of both in-hospital and long-term outcomes, regardless of diabetic status in patients with acute coronary syndrome (ACS). Hence, in this study, we analyzed the incidence of acute hyperglycemia and its impact on subsequent adverse in-hospital outcomes, irrespective of diabetic status, in patients with STEMI undergoing primary PCI. Acute hyperglycemia was observed not only in diabetic but also in non-diabetic patients in a ratio of 25.0% and 65.4%, respectively. Acute hyperglycemia was found to be associated with an increased risk of mortality (5.7% vs. 2.5%; p < 0.001) in both people with diabetes (6.9% vs. 2.9%; p = 0.001) and non-diabetics (4.0% vs. 2.5%; p = 0.028) patients. Prolonged ischemia has been observed to be associated with acute hyperglycemia in both diabetic and non-diabetic patients.

In concordance with our findings, Kim EJ et al. [7] reported acute hyperglycemia in 23.3% it was 8.1% among non-diabetics and 52.0% among diabetic patients. Acute hyperglycemia was found to be an independent predictor of in-hospital mortality with an adjusted hazard ratio of 2.5 [1.26–4.96]. Khalfallah M et al. [16] 16.8% stress hyperglycemia among 660 patients with STEMI, family history of DM, body mass index > 24 kg/m^2^, and cardiogenic shock on admission were reported as the independent predictors of stress hyperglycemia.

In-hospital outcomes and complications such as mortality, cardiogenic shock, and CIN were found to be significantly higher among the stress hyperglycemia group. Qin Y et al. [17] reported acute hyperglycemia in 36.98% of the patient with STEMI, and the area under the curve (AUC) for random blood sugar level was 0.789 [0.759–0.816] for predicting in-hospital death. In addition to random blood sugar, hypertension, DM, age, and fasting blood sugar level were reported to be independent predictors of mortality. Tran HV et al. [18] reported hyperglycemia to be as high as 51.9% of acute myocardial infarction patients at the time of hospital admission, and it was found to be associated with an increased risk of ventricular tachycardia. Their studies have reported the prognostic role of acute hyperglycemia in patients with acute myocardial infarction, with or without DM [6, 8, 19]. Furthermore, the in-hospital mortality rates were compared among different patient groups based on their diabetic and glycemic status. Among non-diabetic normoglycemic patients, the mortality rate was found to be 2.5%. However, non-diabetic hyperglycemic patients exhibited a significantly higher mortality rate of 4.0% (p = 0.028). Even more concerning, diabetic hyperglycemic patients had a substantially higher mortality rate of 6.9% (p < 0.001). In contrast, there was no statistically significant difference in the in-hospital mortality rate for diabetic normoglycemic patients, which remained at 2.9% (p = 0.539). These findings underscore the importance of glycemic control in patients with and without diabetes, as elevated blood glucose levels appear to be associated with increased mortality risk during hospitalization.

As observed in our study, both acute and chronic hyperglycemia is associated with an increased risk of mortality, later higher than the former, after STEMI. However, a typical rise in admission plasma glucose level cannot differentiate between acute hyperglycemia and chronic hyperglycemia due to the major confounding effect of DM and the higher rate of undiagnosed DM, especially in our parts of the world [20]. Hence, the stress hyperglycemia ratio [SHR] proposed by Roberts GW et al. [21] is the ratio of admission plasma glucose level to the estimated average glucose (HbA1c). And in multiple studies, SHR has been reported to be an excellent prognostic marker for patients with acute myocardial infarction [8, 14, 20–25]. Caturano A et al. [26], in their review of the literature, have summarized clinical pathophysiology evidence regarding the cardioprotective effect of strict glycemic control in ACS patients and reported several possible mechanisms for an increased risk of adverse outcomes in hyperglycemic ACS patients. High glucose triggers endothelial NADPH oxidase-2 (NOX2) activation, disrupting the balance between Raf/MAPK-mediated vasoconstriction and PI3K/Akt-induced vasodilation, favoring constriction. Additionally, hyperglycemia induces superoxide overproduction via various mitochondrial electron transport chain mechanisms. This leads to increased infarct size by promoting myocardial cell apoptosis and reducing collateral blood flow. Furthermore, elevated free fatty acid (FFA) levels induce myocardial toxicity during acute ischemia [26]. Several studies have reported the positive effects of tight glycemic control in ACS patients, especially in patients with Type II diabetes [26, 27].

However, HbA1c is not a routine laboratory assessment for STEMI patients in most clinical setups, while random blood glucose and an easy-to-perform and routine laboratory assessment in the emergency room before primary PCI. Despite all its shortcomings, hyperglycemia is a marker for the inflammatory/stress state of the body in an acute situation; it has been found to be associated with prolonged ischemic time and increased risk of adverse clinical outcomes regardless of diabetic status. Even though it can not play a causative role by itself but it can be an attractive choice as a simple prognostic marker for early risk stratification of patients with STEMI.

To our knowledge, this study is based on the largest cohort of STEMI patients from Pakistan, even though some limitations need to be acknowledged, including single-center experience, lack of short- or long-term follow-up, and lack of laboratory assessment of HbA1c for the confirmation of undiagnosed diabetes and differentiation of acute hyperglycemia and uncontrolled chronic hyperglycemia.

## Conclusion

Acute hyperglycemia, regardless of diabetic status, is found to be an independent predictor of in-hospital mortality among patients with STE-ACS undergoing primary PCI. Acute hyperglycemia, along with other significant predictors such as multi-vessel involvement, LVEDP, and Killip class III/IV, can be considered for the risk stratification of these patients.

## Data Availability

Data and material will be available upon request to the corresponding author.

## List of abbreviations

ACS: acute coronary syndrome
PCI: percatenouss coronary intervention
RBS: random plasma glucose
LVEDP: left ventricular end-diastolic pressure
CVD: cardiovascular disease
LMICs: low- and middle-income countries
STEMI: ST-segment elevation myocardial infarction
DALY: disability-adjusted life years
DM: diabetes mellitus
MACE: major adverse cardiovascular events
DAPT: dual antiplatelet therapy
SD: standard deviation
IQR: interquartile range
CVA: cerebrovascular accident
OR: odds ratio
CI: confidence interval
